# Health Risks in Same-Sex Attracted Ugandan University Students: Evidence from Two Cross-Sectional Studies

**DOI:** 10.1371/journal.pone.0150627

**Published:** 2016-03-16

**Authors:** Anette Agardh, Michael Ross, Per-Olof Östergren, Markus Larsson, Gilbert Tumwine, Sven-Axel Månsson, Julie A. Simpson, George Patton

**Affiliations:** 1 Social Medicine and Global Health, Department of Clinical Sciences, Lund University, Malmö, Sweden; 2 Department of Family Medicine and Community Health, University of Minnesota Medical School, Minneapolis, United States of America; 3 Department of Obstetrics and Gynaecology, St Francis Hospital, Nsambya, Uganda; 4 Centre for Sexology and Sexuality Studies, Malmö University, Malmö, Sweden; 5 Centre for Epidemiology and Biostatistics, Melbourne School of Population and Global Health, University of Melbourne, Melbourne, Victoria, Australia; 6 Centre for Adolescent Health, Royal Children’s Hospital, Murdoch Children’s Research Institute, Department of Paediatrics, University of Melbourne, Victoria, Australia; University of Westminster, UNITED KINGDOM

## Abstract

Widespread discrimination across much of sub-Saharan Africa against persons with same-sex sexuality, including recent attempts in Uganda to extend criminal sanctions against same-sex behavior, are likely to have profound effects on this group’s health, health care access, and well-being. Yet knowledge of the prevalence of same-sex sexuality in this region is scarce. This study aimed to systematically examine prevalence of same-sex sexuality and related health risks in young Ugandan adults. We conducted two cross-sectional survey studies in south-western Uganda targeting student samples (n = 980, n = 1954) representing 80% and 72% of the entire undergraduate classes attending a university in 2005 and 2010, respectively. A questionnaire assessed items concerning same-sex sexuality (same-sex attraction/fantasies, same-sex sexual relations), mental health, substance use, experience of violence, risky sexual behavior, and sexual health counseling needs. Our findings showed that same-sex sexual attraction/fantasies and behavior were common among male and female students, with 10–25% reporting having sexual attraction/fantasies regarding persons of the same-sex, and 6–16% reporting same-sex sexual relations. Experiences of same-sex sexuality were associated with health risks, e.g. poor mental health (2010, AOR = 1.5; 95% CI: 1.0–2.3), sexual coercion (2010, AOR 2.9; CI: 1.9–4.6), and unmet sexual health counseling needs (2010, AOR 2.2; CI: 1.4–3.3). This first study of young adults in Uganda with same-sex sexuality found high levels of health needs but poor access to health care. Effective response is likely to require major shifts in current policy, efforts to reduce stigmatization, and reorientation of health services to better meet the needs of this vulnerable group of young people.

## Introduction

Recent efforts to scale-up existing legislation criminalizing same-sex behavior in Uganda have been linked to stigma, discrimination, violence, and persecution. [1]Although the prohibition of same-sex acts existed in Uganda already in 1950, homophobic rhetoric in public discourse became particularly intense during the 2000’s, fuelled by religious and political debates. The Anti-Homosexuality Bill (AHB), first introduced in 2009 and signed into law by President Museveni in February, 2014, sought to legitimize the persecution of persons engaged in same-sex behavior by radically extending the scope of same-sex associated crimes and penalties. Although the AHB was subsequently ruled invalid by the Constitutional Court in Uganda on August 1, 2014, current law stipulates up to 14 years imprisonment for homosexual acts. Homophobic discourse is widespread, and many healthcare practitioners still regard homosexuality as deeply pathological [1].

In homophobic settings, persons who are same-sex attracted face health risks beyond an increased risk for HIV and sexually transmitted infections among men [2]. They also encompass violence and sexual coercion, and especially among women, “corrective rape” [3,4], as well as poor mental health and risky lifestyles involving unsafe sex and alcohol and drug usage. Ross found that high internalized homonegativity in gay and bisexual men in Uganda was associated with increased risk behavior [5]. Nevertheless, fear of disclosure may prevent help seeking for health problems, particularly those related to sexual and reproductive health. Some healthcare workers in Uganda are reluctant to treat persons with same-sex orientation, with reports of blackmailing, denial of services, and discriminatory attitudes among healthcare providers [6,7].

The current political climate in Uganda has created challenges for health promotion campaigns that address risky same-sex practices, leaving most of the health awareness-raising work to interest groups working under the threat of various reprisals—both legal and social [8]. In the absence of information, education, and counseling, misbeliefs and myths are likely to proliferate [9,10]. The problems are compounded by an absence of studies indicating how common same-sex sexuality, broadly defined, might be in Uganda, as well as, the associated health risks. Prevalence studies of same-sex sexuality from other settings in sub-Saharan Africa have primarily focused on men who have sex with men, with evidence obtained from patient populations or respondent-driven sampling [11–13].

The aim of the current study was to examine the prevalence of same-sex sexuality and its links to health risks, including unmet health care needs, in a young adult Ugandan population. To our knowledge, this is the first study of same-sex sexuality that assesses the prevalence of physical and non-physical same-sex behavior and related health risks in a sub-Saharan country where same-sex sexual activity is illegal. Two studies examining same-sex sexuality solely in terms of physical behavior and with a focus on HIV risk were conducted in South Africa [14,15], where same-sex sexual activity is legal, but only one of those studies included females [15].

The current analyses were based on two university-wide surveys conducted in 2005 and 2010 among male and female students at a large public university in south-western Uganda. The surveys were intended to provide information about young adults’ lifestyles and sexual behavior, including experiences of same-sex sexuality. As same-sex “desire”, e.g. attraction and fantasies (internalized behavior), might be more common among young adults than same-sex sexual relations/physical activity (externalized behavior), we purposefully investigated the prevalence of both types of experiences in the intended target group.

## Methods

### Population and setting

#### Participants

Participants for this survey were recruited from Mbarara University of Science and Technology (MUST), a public university in the city of Mbarara in southwestern Uganda. Mbarara is Uganda’s third largest city, and although students attending MUST originate from all over Uganda, the proportion of young adults who attend university in Uganda is small. Thus, among Ugandans aged 20–24, 8.9% were enrolled in post-secondary (higher) education [16].

The entire undergraduate classes in the years 2005 (*n* = 1220 students) and 2010 (*n* = 2706 students) were invited to complete the survey. This encompassed the faculties of Medicine, Science, Development Studies, and Computer Science. The Institutional Ethical Review Committee at MUST granted approval for the study in 2005 and 2010.

### Procedures

#### Survey instrument

A 132-item self-report questionnaire was used. The questions on sexuality, mental health, and substance use were based on previously used validated instruments [17–20]. The questionnaire was pre-tested both individually and in small group discussions with other tertiary students in Mbarara district. The questionnaire was administered in lecture halls during normal class times. The research staff ensured that the room was silent while students were engaged in filling out the questionnaires, so that each person could work in private and not see the neighbor’s answers. The completed questionnaires were to be turned in anonymously in a box in the lecture hall. Very few students left the classroom without turning in a questionnaire.

Students received oral and written information about the purpose of the study and were asked to sign a consent form prior to participation. Anonymity of the participants and confidentiality of the information given were emphasized as part of the procedural description. The same questionnaire was used for both samples, and the procedures regarding administration of the instrument were identical.

### Measures

The questionnaire assessed experience of same-sex sexuality, current mental health, substance use, violence, risky sexual behavior, and unmet needs of sexual health counseling.

Independent measures

*Same-sex sexuality* was defined as being in love with, being sexually attracted to, having fantasies about, or having sexual relations with a person of the same sex. These four same-sex sexuality measures were assessed by the corresponding questions: If you think of the people you have been in love with, what gender were they? If you think of the people you have been sexually attracted to, what gender were they? If you think of the people you have sexually fantasized about, what gender were they? If you think of the people you have had sexual relations with, what gender were they?

The response alternatives were: “always female”; “usually female but sometimes male”; male/female equally”; “usually male but sometimes female”; and “always male”. This was then dichotomized for male respondents as follows: the first alternative was defined as “heterosexual experience” and the latter four as “experience of same-sex sexuality”. Correspondingly, for female respondents, the last alternative was defined as “heterosexual experience” and the prior four as “experience of same-sex sexuality”.

For the purpose of multivariable analysis, an aggregate variable representing internalized same-sex sexuality was created whereby persons were coded as “being sexually attracted to and/or having sexually fantasized about one of the same sex if they reported “same-sex experience” regarding at least one of the two following questions: *If you think of the people you have been sexually attracted to*, *what gender were they*? *If you think of the people you have sexually fantasized about*, *what gender were they*? Being in love with a person of the same sex was not included in this aggregate variable.

#### Dependent measures (health risks)

Mental health was assessed by means of the Hopkins Symptom Check List-25 (HSCL-25). This self-reporting instrument consists of 15 items assessing symptoms of depression and 10 items assessing symptoms of anxiety during the week prior to its administration. In addition, 10 items from the Symptom Checklist-90 (SCL-90) were included for the assessment of symptoms of psychoticism during the previous week. Each item on the HSCL-25 is graded on a four-point scale ranging from [1] “not at all” to [4] “extremely.” The SCL-90 is also a self-reporting instrument whose five-point scale was developed to assess psychiatric symptoms. To attain a homogeneous classification, we rated the 10 psychoticism items in the same way as anxiety and depression, i.e., on a scale of 1 to 4. The HSCL has been widely used in a variety of clinical and nonclinical settings, and its high degree of reliability and validity is well documented [18,19]. Moreover, both HSCL-25 and SCL-90 have been employed and validated in different cultural contexts in Africa, including Uganda [18]. For the purpose of analysis a summary symptom score was derived for each individual. Mental health was dichotomized with poor mental health defined as a summary score above the median.

Substance use was defined in terms of frequent heavy episodic drinking, consumption of alcohol on latest occasion of sexual intercourse, and having ever smoked cannabis. Frequent Heavy Episodic Drinking was assessed by the question “How often do you drink six ‘glasses’ or more on the same occasion?” Responses were categorized based on the responses “daily, or almost daily,” “every week,” “every month”—all of which were coded as “yes”—and “less than once a month” and “never,” which were coded as “no.”

Violence was defined as a positive response to two questions, i.e. experience of physical violence in the last 12 months and experience of sexual coercion [20,21].

Risky sexual behavior was defined as multiple sexual partners in the last 12 months, inconsistent condom use, having accepted money/gift in exchange for sex, and as having paid for sex.

Unmet sexual health *counseling needs* were defined as a positive response to the question: Have you experienced any sexual health problem in the last three months that made you think you needed to see a counselor, but you refrained from doing so?

### Statistical Analysis

Statistical analysis was conducted using Stata Version 12 (StataCorp. College Station, TX, USA). The sample size was constrained to all students enrolled at the university in 2005 and 2010, and thus no formal sample size calculation was performed. Descriptive statistics were presented to describe the characteristics of the study population, including prevalence of same-sex sexuality. Prevalences of same-sex sexuality within each survey year were compared between males and females using the chi-square test.

Logistic regression analyses, adjusted for age and sex, were performed to calculate the adjusted odds ratios (AOR) and 95% confidence intervals (CI) for the association in each sample between two measures representing same-sex sexuality and the health risks represented by poor mental health, substance use, violence, risky sexual behavior, transactional sex, and unmet sexual health counseling needs. Cases with missing data were excluded from the analyses. The proportion of missing responses for each variable varied from 14–24%, with the highest proportion observed for the question concerning same-sex sexual relations.

## Results

In 2005, 980 students (80% of those enrolled) participated in the survey; in 2010 the figure was nearly double, i.e. 1,954 participants (72% of those enrolled) due to increased enrollment at the university.

Table 1 shows the socio-demographic characteristics of the students surveyed. The proportion of females and younger participants were higher in the 2010 sample (females, 35% in 2005 and 44% in 2010; aged less than 24 years, 66% and 72%). Almost half of the students came from rural areas, a percentage that was higher for males in both survey years (51% for 2005 and 49% for 2010) than for females (31% and 40%). In 2005, 75% of the total student population came from families in which the head of household had attended secondary school or beyond; in 2010, the corresponding percentage (71%) was similar.

**Table 1:** Characteristics of university students in Uganda in 2005 (n = 980) and 2010 (n = 1954).

	2005						2010					
	All		Male		Female		All		Male		Female	
	n	%	n	%	n	%	n	%	n	%	n	%
*Sex*												
Male	633	64.6					1087	55.6				
Female	347	35.4					867	44.4				
*Age*												
Younger < 24	628	65.6	378	60.6	250	75.1	1346	71.7	708	67.4	638	77.2
Older ≥ 24	329	34.4	246	39.4	83	24.9	531	28.3	343	32.6	188	22.8
Missing	(23)		(9)		(14)		(77)		(36)		(41)	
*Area of origin*												
Rural	424	43.7	318	50.6	106	31.0	869	44.9	526	48.8	343	39.9
Urban/ peri–urban	546	56.3	310	49.4	236	69.0	1067	55.1	551	51.2	516	60.1
Missing	(10)		(5)		(5)		(18)		(10)		(8)	
*Educational level of head of household*												
≤ Primary school	235	25.5	186	31.0	49	15.2	518	27.3	329	31.1	189	22.5
>Primary school	688	74.5	414	69.0	274	84.8	1382	70.7	730	68.9	652	77.5
Missing	(57)		(33)		(24)		(54)		(28)		(26)	

### Prevalence of internalized and externalized same-sex sexuality

Table 2 presents the prevalence of same-sex sexuality among students in terms of internalized and externalized behavior. Prevalences of same-sex sexuality were high in this sample of university students. Approximately one in three reported having been in love with someone of the same sex. Almost one in five reported being attracted to someone of the same sex and one in ten a sexual relationship with someone of the same sex. Females consistently had higher prevalences of all same-sex measures compared to males, in both samples. All same-sex prevalences were somewhat lower in the 2010 sample across both genders.

**Table 2:** Prevalence of same-sex sexuality by gender among university students in Uganda, 2005 (n = 980) and 2010 (n = 1954).

	**Gender**				
**2005**	**Male (N = 633)**		**Female (N = 347)**		***p*-value**
	**Frequency (n)**	**% (95% CI)**	**Frequency (n)**	**% (95% CI)**	
Been in love with someone of the same sex	167	30 (26–34)	123	41 (36–47)	0.001
Sexually attracted to or sexually fantasized of someone of same sex	85	15 (12–18)	72	25 (20–30)	<0.001
Sexual relation with someone of the same sex	39	8 (6–11)	34	16 (11–21)	0.002
	**Gender**				
**2010**	**Male (N = 1087)**		**Female (N = 867)**		***p*-value**
	**Frequency (n)**	**% (95% CI)**	**Frequency (n)**	**% (95% CI)**	
Been in love with someone of the same sex	232	25 (22–28)	267	35 (32–39)	<0.001
Sexually attracted to or sexually fantasized of someone of same sex	100	10 (8–12)	147	19 (17–22)	<0.001
Sexual relation with someone of the same sex	52	6 (5–8)	59	10 (7–12)	0.015

### Relationships between same-sex sexuality and poor health outcomes

Table 3 shows the extent of association between a range of health risks and two measures of same-sex sexuality by sample.

**Table 3:** Associations (Prevalence and Adjusted OR, 95% CI) between poor mental health, substance abuse, violence, risky sexual behavior, unmet sexual health counseling needs and experience of same-sex sexuality in 2005 and 2010.

	2005						2010					
	Sexually attracted to or fantasized of someone of the same sex			Sexual relation with someone of the same sex			Sexually attracted to or fantasized of someone of the same sex			Sexual relation with someone of the same sex		
	YES (%)	NO (%)	OR (95% CI)*	YES (%)	NO (%)	OR (95% CI)*	YES (%)	NO (%)	OR (95% CI)*	YES (%)	NO (%)	OR (95% CI)*
**Poor Mental health**	69.2	46.7	2.6 (1.8–3.7)	75.3	49.9	3.1 (1.8–5.3)	57.9	48.4	1.5 (1.1–1.9)	61.0	51.0	1.5 (1.0–2.3)
**Substance use**												
Frequent heavy episodic drinking	26.5	17.4	2.1 (1.3–3.8)	32.9	20.3	2.2 (1.2–4.0)	37.1	26.6	1.6 (1.0–2.6)	41.9	29.4	1.7 (1.02–2.7)
Consumed alcohol on latest occasion of sexual intercourse	54.6	32.2	2.6 (1.5–4.6)	63.2	33.6	3.5 (1.7–7.1)	23.7	15.9	1.9 (1.2–3.1)	27.9	16.2	2.1 (1.2–3.9)
Ever smoked cannabis	13.0	5.7	2.8 (1.5–5.1)	26.6	6.7	5.9 (3.0–11.7)	5.5	4.9	1.6 (0.6–2.3)	11.7	5.4	2.3 (1.2–4.6)
**Violence**												
Experience of physical violence in last 12 months	12.7	9.1	1.5 (0.8–2,5)	12.9	10.4	1.5 (0.7–3.2)	14.0	9.8	1.7 (1.0–2.9)	17.4	10.9	1.5 (1.03–2.3)
Experience of sexual coercion	59.4	26.3	3.8 (2.6–5.7)	69.6	31.9	4.6 (2.7–8.0)	46.3	26.7	2.1 (1.6–2.9)	58.8	30.5	2.9 (1.9–4.6)
**Sexual behavior**												
Multiple sexual partners in last 12 months	44.1	34.1	1.7 (1.0–2.9)	48.5	34.7	2.1 (1.01–4.4)	45.1	36.2	1.8 (1.2–2.8)	46.6	36.7	1.8 (1.1–3.2)
Inconsistent condom use	22.2	18.1	1.3 (0.7–2.3)	37.2	16.7	2.9 (1.5–5.7)	39.1	29.2	1.4 (0.9–2.0)	31.0	29.6	1.1 (0.5–1.6)
Accepted money/gift in exchange of sex	16.7	10.2	1.8 (1.0–3.2)	22.8	11.8	2.3 (1.2–4.7)	14.3	7.1	1.9 (1.1–3.1)	18.9	8.5	2.0 (1.1–3.7)
Paid for sex	31.2	16.7	2.4 (1.5–4.0)	38.0	21.6	2.5 (1.3–4.8)	24.2	13.9	2.8 (1.8–4.2)	29.0	16.9	2.5 (1.5–4.1)
**Sexual health services**												
Unmet sexual health counseling need	34.0	18.9	2.2 (1.5–3.4)	49.2	21.3	3.6 (2.1–6.3)	35.4	25.2	1.5 (1.1–2.1)	46.3	28.1	2.2 (1.4–3.3)

* adjusted for age and gender

On virtually all main measures of health risks, i.e. poor mental health, substance use, violence, and risky sexual behavior, those reporting experience of same-sex sexuality had greater odds of at least one health risk, compared with those with no same-sex sexuality experiences. They also had higher odds unmet sexual health counseling needs. Moreover, those with same-sex sexuality had generally similar levels of associated health risks in both samples, regardless of whether they had reported same-sex attraction or same-sex sexual relations. Same-sex sexuality was associated with experiences of sexual coercion in both samples, but with physical violence solely in 2010. Also, a significant association between same-sex sexual relations and inconsistent condom use was seen solely in the 2005 sample.

## Discussion

This is the first study of the prevalence of same-sex sexuality, broadly defined, from a sub-Saharan country where homosexuality is illegal, using data obtained form the reported experiences of young male and females adults. Same-sex attraction and behavior were common in these two samples of young Ugandan adults. One in five reported being sexually attracted to someone of the same sex and one in ten had a history of a sexual relationship with someone of the same sex. Across all health indicators included in this survey, including risky sexual behavior, experience of sexual coercion and violence, poor mental health and risky substance use, same-sex attracted young adults in Uganda are faring poorly. Many of the negative health consequences are likely to be a result of stigmatization and having to hide one’s sexual orientation. Given the size of this subgroup, the health risks currently indicated have the potential to undermine Uganda’s progress in HIV control.

The findings suggest that health service systems are limited in their capacity to meet the health needs of this group. Despite their relative affluence and higher levels of education, almost half reported unmet sexual health counseling needs. Even though the most severe punishments proposed in the 2009 Anti-Homosexuality Bill (AHB) [22] have subsequently been retracted, the continuing political and social hostility towards same-sex sexuality in Uganda is both likely to discourage young persons with same-sex sexuality from seeking healthcare and diminish effective responses from many healthcare providers.

Homosexual behavior is currently demonized and stigmatized in Uganda; however, the prevalence of same-sex sexual relations was about the same in both samples surveyed 5 years apart, and similar to the prevalences reported in two recent studies from South Africa, where same-sex practices are legal [14,15]. Although the question of how illegality influences poor health outcome is interesting, the two South African studies used somewhat different methodology and focused primarily on the association between same-sex practices and male HIV risk. Our prevalences ranging from 6–8% in males and 10–16% in females are similar to other studies, i.e. 5.4% lifetime prevalence in males aged 18–49,[7] and 4.9% prevalence in males and females aged 18–24 [14] thus lending support to the validity of the current findings, as well as to the notion that same-sex sexuality experiences are not uncommon in sub-Saharan Africa. A study of sexual behavior among university students in Ethiopia found similarly that 6% had had intercourse with same-sex partners [23]. Our prevalence estimates of same-sex “desires”, such as being in love with, sexual attraction towards, and fantasies about persons of the same-sex, are also similar to those from other high, middle, and low-income countries [14,15,24–27]. Nevertheless, the prevalences obtained across all three measures of same-sex sexuality in the 2010 sample were consistently lower than those obtained in 2005. Although the reason(s) for the lower prevalences is unknown, the highly negative attitudes about same-sex sexuality that have been widely publicized in the past few years might have contributed to reluctance among respondents to report experiences of same-sex sexuality.

This is one of the very few epidemiological studies of same-sex sexuality conducted in sub-Saharan Africa that utilizes data drawn from a large sample of healthy young adults (male and female university students), in contrast to previous studies conducted at health care clinics or within the “gay community”. The large sample size, including the high response rates of 72–80% of those enrolled at a major university in Uganda at two points in time, 2005 and 2010, and the comprehensive nature of the survey instrument allow a unique opportunity to explore issues related to same-sex sexuality in a non-clinical sample. A further strength of the current study is the inclusion of a broad range of potentially related health consequences.

This study has several limitations. Firstly, although the achieved sample was similar to the enrolled student body in terms of gender distribution, the sample’s representativeness regarding other background characteristics of the enrolled student body is unknown. It is however unlikely that non-participants (those who were not in class that day) systematically differed, in that no pre-announcement of the survey was given. Nevertheless, university students are likely to be a highly selected group, especially in Uganda where so few young adults attain a higher education. Thus, the representativeness of the sample with regard to Ugandan youth in this age group, as well as other age groups in Uganda might be limited. Moreover, university students may have greater access to health information and to social networks than the general population, and the impact of this on the current findings is unknown. Another important limitation is the cross-sectional nature of the study design, whereby causality between the relationships assessed cannot be established. While the two samples were collected five years apart using identical methodology and with a high proportion of eligible respondents, there may be biases relating to inclusion or failure to respond that we are not aware of. Although the general participation rate was quite high (at least compared with other voluntary surveys using questionnaires, targeting healthy and relatively unselected populations) at 72–80% of the full target group, nonresponse regarding the questions assessing experience of same-sex sexuality ranged between 14–25%. Non-response to any of the same-sex items could have been due to perceived irrelevance or reluctance to report socially undesirable behavior. Moreover, due to social undesirability, students might have underreported experience of same-sex sexuality, thus leading to misclassification. Although the cross-cultural validity of some of the survey items, i.e. “being in love with someone of the same sex”, might be questioned, pre-testing of the survey instrument in small focus group discussions indicated that there was a general consensus about the underlying concepts. Although the questionnaire was administered in the classroom to enhance participation rates, it is possible that some participants did not feel entirely comfortable in this setting, which might have led to non-response or socially desirable responses.

Mental health and substance use correlates of having a heavily stigmatized and criminalized sexual orientation appeared to be somewhat less in the most recent sample. Nevertheless, in the absence of additional measurement points in time, it is unknown whether these differences represent random fluctuations, actual differences in the two samples or their experiences, or the influence of processes/events occurring during the period from 2005 to 2010. For example, despite the increasingly negative political climate towards same-sex sexuality during the five years that have elapsed between the two data collections, students in 2010 may also have had a wider opportunity to obtain unbiased and accurate information about sexuality due to the initiation of a student peer-education intervention introduced at MUST in 2005, which targeted sexual and reproductive health and rights. Thus, the non-significant associations between same-sex behavior and inconsistent condom use found in 2010 may reflect increased awareness about risky sexual practices due to the intervention. Interestingly, the prevalence of engaging in transactional sex, which is also associated with sexual relations with the same sex, seems not to have changed over time.

## Conclusion

The results from this study in Uganda, indicate that young adults with same-sex sexuality face major health challenges, thus calling for continuing action in terms of public health response, media attention, and political advocacy for this group. The fact that this group also has unmet health counseling needs is a considerable part of the challenge. In the current political environment of Uganda, where persons with same-sex sexuality continue to face hostility and persecution, their risk for adverse health consequences including HIV/AIDS due to reluctance to seek health care urgently needs to be addressed. Better health for persons with same-sex sexuality will also require intensified research efforts, as well as a solid commitment from policy makers and health care givers.

## Supporting Information

S1 Questionnaire (PDF)
