# Supplementary material for: Health Risks in Same-Sex Attracted Ugandan University Students: Evidence from Two Cross-Sectional Studies
Source: PLoS One. 2016 Mar 16;11(3):e0150627. doi: 10.1371/journal.pone.0150627 (PMC4794142; doi:10.1371/journal.pone.0150627)
Supplement: S1 Questionnaire — (PDF) [file pone.0150627.s001.pdf]

S1 QUESTIONNAIRE

Your personal code (Choose a combination of three numbers between 1-9 and three letters (write in capital form), e.g. 6AG14P. If you participated last year use the same code if you remember it. Copy this code to the separate sheet that you have received, and save it for the planned follow-up survey.

Personal code |___|___|___|___|___|___|

Questionnaire no |___|___|___|___|

Date |___|___|___|___|___|___|

For some questions there is a frame where you can fill in your answer

1. BACKGROUND

Growing up

(1) Your age in years |___|___|

(2) Sex:

☐ Male
☐ Female

We would first like to ask you about your family background and your life when you grew up.

(3) Where did you grow up (most of the time=most of your life years)

☐ Rural area
☐ Urban area
☐ Periurban or small town (in the periphery of a big city or in a small town)

(4) Which adults did you live with most of the time (most of the years) when you grew up?
Tick one

☐ My mother
☐ My father
☐ My mother and my father
☐ Other

(5) The head of the household when you grew up, what occupation did he or she have?

[ ]

(6) The head of the household when you grew up, what educational level did he or she have?

☐ Not finished primary school
☐ Completed primary school
☐ Completed secondary school
☐ Post secondary school
☐ College
☐ University education
☐ Other

(7) When you went to primary school, was it mainly:

☐ In mixed sex school (boys and girls)
☐ Single sex schools

(8) When you went to secondary school, was it mainly:

a) 0 level
☐ In mixed sex schools
☐ Single sex schools

b) A level
☐ In mixed sex schools
☐ Single sex schools

(9) Did you go to boarding school?

☐ Yes, 1-3 years
☐ Yes, >3 years
☐ No, I did not go to boarding school

(10) What was the main religion in the family where you grew up?

☐ Catholic
☐ Protestant
☐ Moslem
☐ Pentecostal
☐ Seventh Day Adventists
☐ Orthodox
☐ Other

(11) What role did religion play in your family when you grew up?

☐ Religion played a big role
☐ Religion was relatively important
☐ Religion was not so important
☐ Religion was not important at all

2. BEING A UNIVERSITY STUDENT

Now we would like to ask some questions about your current life as a university student

(12) In which study year are you currently?

☐ First year
☐ Second year
☐ Third year
☐ Fourth year
☐ Fifth year

(13) How are your studies going?

☐ My studies are going well
☐ My studies are going reasonably well
☐ My studies are not going as well as I wish

(14) In which faculty are your studies mainly?

☐ Faculty of medicine
☐ Faculty of developmental studies
☐ Faculty of science
☐ Other

(15) What are the main difficulties for you in getting the study results you want?
Tick several if you wish

☐ Limited literature access (including library)
☐ Limited access to IT (information technology): computers, internet etc
☐ Limitations in the teaching (faculty, teaching time or methods etc)
☐ Financial
☐ Accommodation and reading space (privacy, calm etc)
☐ Social activities take too much time
☐ Lack of interest in studies
☐ Interference from fellow student
☐ Other

3. SOCIAL RELATIONS, PARTICIPATION AND TRUST (SOCIAL CAPITAL)

Now we would like to ask you some questions about your social life.

(16) Make a decision about the following statements

a) Most people would take advantage of you if they had an opportunity

☐ I do not agree at all
☐ I do not agree
☐ I agree
☐ I agree completely

b) Most people try to be fair

☐ I do not agree at all
☐ I do not agree
☐ I agree
☐ I agree completely

c) You can trust most people

☐ I do not agree at all
☐ I do not agree
☐ I agree
☐ I agree completely

d) You cannot be careful enough when dealing with other people

☐ I do not agree at all
☐ I do not agree
☐ I agree
☐ I agree completely

(17) What level of confidence do you have for the following institutions?
Tick one box for each of the institutions

	Very high	Fairly high	Not so high	Low	No opinion
a. Health care system	☐	☐	☐	☐	☐
b. School system	☐	☐	☐	☐	☐
c. Police	☐	☐	☐	☐	☐
d. Judicial system	☐	☐	☐	☐	☐
e. National parliament	☐	☐	☐	☐	☐
f. District politicians	☐	☐	☐	☐	☐
g. Local politicians	☐	☐	☐	☐	☐
h. Mass media, TV, newspaper	☐	☐	☐	☐	☐

(18) Have you during the last couple of months: *Tick as many boxes as you need!*

☐ Participated in a course at your work place
☐ Participated in a course in your leisure time
☐ Participated in a union meeting
☐ Visited a theatre/the movies
☐ Visited an art exhibition
☐ Participated in a religious gathering
☐ Visited a sport event
☐ Written a letter to an editor of a newspaper/magazine
☐ Participated in a demonstration of any kind
☐ Visited a public event, e.g. a night club, dance event or similar
☐ Participated in a larger family gathering
☐ Been to a private party
☐ None of the above

(19) During to your social activities, how many of the other participants in those activities are of the same background (e.g. sex, education or country of origin) as your own? *Tick one box*

☐ All the other participants in those activities have the same background as my own
☐ Most of the other participants in those activities have the same background as my own
☐ About half of the participants in those activities have the same background as my own
☐ Most of the other participants in those activities have a different background than my own
☐ All the other participants in those activities have a different background than my own

(20) Does a person's background (e.g. sex, education or country of origin) affect your level of trust for them, for example the credibility for what they say about different things? *Tick one box*

☐ I only trust persons with the same background as my own
☐ I trust persons with the same background as my own rather more than others
☐ I trust persons with the same background as my own a bit more than others
☐ I trust persons with the same background as myself equally as much as others
☐ I trust persons with the same background as myself less than others

(21) How well do your common opinions and values match other persons of the same background (e.g. sex, education or country of origin) as your self? *Tick one box*

☐ All my opinions and values are the same as other persons' with the same background as my own
☐ Most of my opinions and values are the same as other persons' with the same background as my own
☐ About half of my opinions and values are about the same as other persons' with the same background as my own
☐ Most of my opinions and values differ compared with other persons' with the same background as my own

(22) Have you at any time during the last twelve months been exposed to any threats or threats of violence which were so dangerous or serious that you became scared?

☐ Yes
☐ No

(23) Have you at any time during the last twelve months been a victim of physical violence?

☐ Yes
☐ No

4. LIFE STYLE

We would now like to ask you some questions about your life today.

(24) Do you smoke?

☐ Yes
☐ No → **If no, continue to question 26**

(25) Do you smoke daily?

☐ Yes → **If yes, continue to question 29**
☐ No

(26) Have you earlier smoked daily during at least 6 months?

☐ Yes
☐ No → **If no, continue to question 30**

(27) How long ago did you stop smoking daily?

|___|___|___| months

(28) How old were you when you stopped smoking daily?

|___|___| years

(29) Would you like to stop smoking?

☐ No
☐ Yes
☐ I am not sure
☐ I do not smoke at present

Here are some questions about your alcohol consumption habits

By one "glass" we mean:

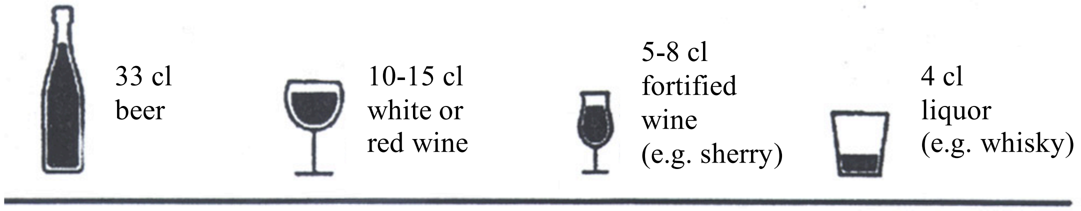

(30) How often have you consumed alcohol during the last twelve months?

☐ 4 times/week or more often
☐ 2-3 times/week
☐ 3-4 times/month
☐ Once a month or more seldom
☐ Never → **If yes, continue to question 34**

(31) How many "glasses" (see example) do you drink during a typical day when you drink alcohol?

☐ 1-2
☐ 3-4
☐ 5-6
☐ 7-9
☐ 10 or more

(32) How often do you drink six "glasses" or more on the same occasion?

☐ Daily, or almost daily
☐ Every week
☐ Every month
☐ More seldom than once a month
☐ Never

(33) How often, during the last twelve months have you consumed so much alcohol that you became drunk?

☐ Daily, or almost daily
☐ A few times per week
☐ Once a week
☐ 2-3 times per month
☐ Once a month
☐ A few times during six months
☐ More seldom or never

(34) Have you ever smoked cannabis/marihuana?

☐ No
☐ Yes, during the last month
☐ Yes, during the last year
☐ Yes, more than one year ago

(35) What was your level of physical activity in your leisure time during the last twelve months?

☐ **Regular physical exercise or training**
(e.g. running, swimming, playing tennis or similar activities at least three times a week and for at least 30 minutes each time)

☐ **Regular physical exercise in your leisure time**
(By "regular" means 1-2 times per week, at least 30 minutes each time, e.g. running, swimming, playing tennis etc)

☐ **Some physical exercise in your leisure time**
(You walk, cycle, or move about during at least 2 hours per week, included walk to courses, to town, etc)

☐ **Physically inactive leisure time**
(You spend most of your time by reading, watching TV, sitting talking or similar activities. You walk, cycle or move about less than 2 hours per week)

5. RELATIONS, LOVE AND SEXUALITY

The following personal questions are demanding to answer, and quite important.

(36) Have you ever had a girl/boy friend? By girl/boy friend, I mean someone to whom you were sexually or emotionally attracted and whom you 'dated'.

☐ Yes
☐ No

(37) How many girl/boy friends have you had?

|___|___|___|

(38) Do you have a girl/boyfriend at the moment?

☐ Yes
☐ No

(39) For how long have you had your present girl/boyfriend?

|___|___|___| **months**

(40) Which contraceptive methods have you heard of?

☐ Contraceptive pill
☐ Condom
☐ Withdrawal
☐ Norplant
☐ Other

(41) Have you ever had sexual intercourse?

☐ Yes
☐ No → **If no, continue to question 52**

(42) At what age did you have sexual intercourse for the first time?

|___|___| **years**

(43) How many sexual partners have you had during the last twelve months?

|___|___|

(44) How many sexual partners have you had altogether?

|___|___|

(45) Did you use any *method* for avoiding sexually transmitted diseases on your latest occasion of sexual intercourse?

☐ No
☐ Yes, condom
☐ Yes, other

(46) Did you use any *method* for avoiding pregnancy on your latest occasion of sexual intercourse?

☐ No
☐ Yes, condom
☐ Yes, contraceptive pill
☐ Yes, other method

(47) How often do you use a condom with a new sexual partner?

☐ Always

☐ Often
☐ Sometimes
☐ Never
☐ Does not apply to me

(48) How do you compare the degree of pleasure using a condom during intercourse, with not using one?

☐ No difference
☐ Less pleasure with a condom
☐ More pleasure with a condom

(49) I intend to use a condom whenever I have intercourse with

☐ Any new sex partner
☐ A casual partner
☐ A regular partner
☐ Any partner

(50) I am satisfied with my ability to use a condom correctly

☐ Yes
☐ No

(51) I believe I can persuade a new sex partner to use a condom

☐ Yes
☐ No

(52) My friends at the university always use a condom with a new partner

☐ Yes
☐ No

(53) My friends at the university would have difficulty demanding condom use with a new partner

☐ Yes
☐ No

If the answer is no on question no 41, please continue to question no 56

(54) Had you been drinking any alcohol on your latest occasion of sexual intercourse?

☐ Yes
☐ No

(55) How often do you drink alcohol when having sexual intercourse?

☐ Always, or almost always
☐ More often than on 50% of the occasions
☐ About on 50% of the occasions
☐ More seldom than on 25% of the occasions
☐ Almost never, or never

(56) If you think of the people you have been in love with, what gender were they?

☐ Always female
☐ Usually female but sometimes male
☐ Male / female equally
☐ Usually male but sometimes female
☐ Always male

(57) If you think of the people you have been sexually attracted to, what gender were they?

☐ Always female
☐ Usually female but sometimes male
☐ Male / female equally
☐ Usually male but sometimes female
☐ Always male

(58) If you think of the people you have sexually fantasised about, what gender were they?

☐ Always female
☐ Usually female but sometimes male
☐ Male / female equally
☐ Usually male but sometimes female
☐ Always male

(59) If you think of the people you have had sexual relations with, what gender were they?

☐ Always female
☐ Usually female but sometimes male
☐ Male / female equally
☐ Usually male but sometimes female
☐ Always male

It sometimes happens that people are forced or drawn into sexual acts against their will. We now ask some questions on that subject. We will first ask whether you have ever *been forced* by violence or some other means into one or more of the sexual acts below.

People can also take part in sexual acts that they do not wish to take part in without being directly forced to do so. We will therefore also ask you whether you have ever taken part in any of these acts *against your will,* e.g. to keep the peace at home.

(60) People can sometimes be *forced into sexual acts.* Have you ever been *forced* into any of the following? *Mark as many boxes as you need!*

☐ You have been forced to show your sexual organ
☐ Someone has forced you to let them touch your sexual organ
☐ Someone has forced you to let them suck or lick your sexual organ
☐ Someone has forced you to let them show you their own sexual organ
☐ You have been forced to watch someone masturbate
☐ You have been forced to masturbate someone
☐ You have been forced to take part in oral sex or to lick someone's sexual organ
☐ You have been forced to take part in sexual intercourse with the penis in the vagina or someone has inserted an object into your vagina
☐ You have been forced to pose for a sex photo or sex film
☐ You have not been *forced* into any of these

If you have been *forced* into any of these:

(61) Has this happened once or a number of times?

☐ Once
☐ A number of times

(62) How old were you the first time it happened?

Age: |___|___|

(63) Is it still going on?

☐ No
☐ Yes

(64) Which person or persons forced you?
Mark as many as you need

☐ Biological relative(s) or household member(s)
☐ Other household member(s)
☐ Biological relative(s) who was not a household member(s)
☐ Other person(s)

☐ Sexual partner(s) of your own choice
→ ☐ Casual/occasional partner(s)
→ ☐ Regular partner(s)

☐ Other
→ ☐ Casual/occasional partner(s)
→ ☐ Regular partner(s)

(65) Have you ever *accepted* money, gift or some other form of compensation as payment for sexual relations with someone?

☐ No, it has never happened → go to question no 71
☐ No, but I have fantasised about it → see above
☐ Yes

If yes:

(66) When was the last time it happened?

☐ Less than a week ago
☐ Less than 30 days ago
☐ Less than 12 months ago
☐ Less than five years ago
☐ Less than ten years ago
☐ Ten years or more ago

(67) What was the age of the person from whom you last accepted money or some other form of compensation as payment for sex?

☐ About the same age as my own
☐ Older than me (but not more than ten years older)
☐ Older than me (more than ten years older)

(68) What was the gender of the person from whom you last accepted money or some other form of compensation as payment for sex?

☐ Male
☐ Female

(69) How many times have you accepted money or some other form of compensation as payment for sex?

|___|___| times - *Do you not remember exactly, state the approximate number!*

(70) In what situation did this occur?
Mark the alternative that applies best

☐ I had work but needed more money
☐ I needed work and needed money
☐ I needed money for drugs
☐ I couldn't resist the money/compensation offered
☐ I needed money to be able to study
☐ I was active as a commercial sex worker
☐ Other

(71) Have you ever paid, given a gift or otherwise compensated another person for sexual activities?

☐ Yes
☐ No

6. YOUR HEALTH

(72) How do you classify your current health in general?

☐ Very good
☐ Good
☐ Fair
☐ Bad
☐ Very bad

(73) How tall are you?

|___|___|___| centimetres

(74) How much do you weigh?

|___|___|___| kilograms

Have you during the last 14 days had any of the symptoms or discomfort mentioned below?
Tick one alternative for each symptom / discomfort mentioned below

	not at all	a little	quite a bit	extremely
(75) Ache in shoulders or neck	☐	☐	☐	☐
(76) Back pain, back ache, hip pain or sciatica	☐	☐	☐	☐
(77) Ache or pain in hands, elbows, legs or knees	☐	☐	☐	☐
(78) Headache or migraine	☐	☐	☐	☐
(79) Anxiety or worry	☐	☐	☐	☐
(80) Fatigue	☐	☐	☐	☐
(81) Suffer from insomnia (lack of sleep)	☐	☐	☐	☐
(82) Eczema or rash	☐	☐	☐	☐
(83) Tinnitus (ringing sensation in the ears)	☐	☐	☐	☐
(84) Incontinence (cannot hold urine)	☐	☐	☐	☐
(85) Repeated stomach-/intestine problems	☐	☐	☐	☐

Here is a list of problems that people can get. For each one we ask you how much that problem has bothered or distressed you during the last week, including today.
Tick one alternative for each problem mentioned below

	not at all	a little	quite a bit	extremely
(86) Worrying too much about things	☐	☐	☐	☐
(87) Feeling so restless you can't sit still	☐	☐	☐	☐
(88) The idea that something serious is wrong with your body	☐	☐	☐	☐
(89) Feeling no interest in things	☐	☐	☐	☐
(90) Heart pounding or racing	☐	☐	☐	☐

	not at all	a little	quite a bit	extremely
(91) Feeling lonely even when you are with people	☐	☐	☐	☐
(92) Poor appetite	☐	☐	☐	☐
(93) Feeling that familiar things are strange or unreal	☐	☐	☐	☐
(94) The idea that something is wrong with your mind	☐	☐	☐	☐
(95) Feeling low in energy and slowed down	☐	☐	☐	☐
(96) Feeling fearful	☐	☐	☐	☐
(97) Having thoughts that are not your own	☐	☐	☐	☐
(98) Blaming yourself for things	☐	☐	☐	☐
(99) Suddenly scared for no reason	☐	☐	☐	☐
(100) The idea that you should be punished for your sins	☐	☐	☐	☐
(101) Feeling blue	☐	☐	☐	☐
(102) Difficulty falling asleep or staying asleep	☐	☐	☐	☐
(103) Other people being aware of your private thoughts	☐	☐	☐	☐
(104) Feeling everything is an effort	☐	☐	☐	☐
(105) Spells of terror and panic	☐	☐	☐	☐
(106) Having thoughts about sex that bother you a lot	☐	☐	☐	☐
(107) Crying easily	☐	☐	☐	☐
(108) Trembling	☐	☐	☐	☐
(109) Never feeling close to another person	☐	☐	☐	☐
(110) Thoughts of ending your life	☐	☐	☐	☐
(111) Feeling pushed to get things done	☐	☐	☐	☐
(112) The idea that someone else can control your thoughts	☐	☐	☐	☐
(113) Feelings of worthlessness	☐	☐	☐	☐
(114) Nervousness and shakiness inside	☐	☐	☐	☐
(115) Hearing voices that other people do not hear	☐	☐	☐	☐
(116) Feeling of being trapped or caught	☐	☐	☐	☐
(117) Feeling hopeless about the future	☐	☐	☐	☐
(118) Feeling lonely	☐	☐	☐	☐
(119) Loss of sexual interest or pleasure	☐	☐	☐	☐
(120) Feeling tense or keyed up	☐	☐	☐	☐

(121) Have you at any time during the last three months, due to your own health problem or illness

No	Yes	
☐	☐	been admitted to hospital?
☐	☐	visited a doctor at a hospital, health care center or other health care facility?
☐	☐	visited a nurse?

(122) Have you, at any time during the last three months experienced a health problem, and thought that you needed to visit a doctor but abstained from seeking health care?

☐ Yes several times
☐ Yes
☐ No

(123) What was the main reason for not seeking medical treatment?
You can only tick one alternative

☐ The health problem disappeared
☐ Did not have enough money
☐ Waiting time was to long
☐ Do not think I can get any help
☐ Did not succeed to get in touch with a doctor
☐ Did not know about any good doctor
☐ Did not have time
☐ Wanted to wait for a while
☐ Other reason

(124) Have you, at any time during the last three months experienced a sexual health problem, and thought that you needed to visit a counsellor but abstained from seeking health care?

☐ Yes several times
☐ Yes
☐ No

(125) What was the reason / reasons for not seeking counselling services?
You can only tick one alternative

☐ The health problem disappeared
☐ Did not have enough money
☐ Waiting time was to long
☐ Do not think I can get any help
☐ Did not succeed to get in touch with a counsellor
☐ Did not know about any good counsellor in Mbarara
☐ Did not feel confident with existing counselling services

(126) Have you ever heard about the peer education project at MUST?

☐ Yes
☐ No

Have you participated in any of the following activities in the peer education project?

(127) Discussions about sexual health

☐ Yes
☐ No

(128) Visited the peer project house

☐ Yes
☐ No

(129) Watched a play conducted by peer educators

☐ Yes
☐ No

7. IMPROVING LIFE SKILLS – HOW?

(130) Can you give an example of an improved life skill that would help you managing your life in a positive direction:

+ +

(131) Has any major change in your life occurred since May 2006 (previous assessment) e.g. marriage, death of a significant, other loss of property, legal problems economic problems?

(132) Has anything happened since May 2006 that has had a major effect on your attitude towards life, towards sexual relationships, towards your thoughts about the future?
